# Extraplanar ultrasound-guided multi-tract percutaneous nephrolithotomy: a retrospective comparative study in patients with complex nephrolithiasis

**DOI:** 10.3389/fruro.2026.1713534

**Published:** 2026-02-06

**Authors:** Geng-Geng Wei, Kristine J. S. Kwan, Yu Yang, Qing-Shan Yang, Zhen-Quan Lu, Lin Xiong, Xiang Xu

**Affiliations:** 1 Department of Urology, The University of Hong Kong – Shenzhen Hospital, Shenzhen, Guangdong, China; 2 Department of Surgery, Fudan University Pudong Medical Center, Shanghai, China; 3 Shenzhen Clinical Research Center for Urology and Nephrology, Peking University Shenzhen Hospital, Shenzhen, China

**Keywords:** percutaneous nephrolithotomy, renal calculi, staghorn calculi, ultrasound surgical procedures, urolithiasis

## Abstract

**Purpose:**

This study aimed to compare the efficacy and safety of extraplanar ultrasound (USG) guidance with the conventional technique in multi-tract percutaneous nephrolithotomy (PCNL) for managing complex renal stones (CRS).

**Materials and methods:**

A retrospective analysis was conducted on 91 patients diagnosed with CRS treated with multi-tract PCNL between May 2017 and December 2020. Patients were divided into the conventional USG group and the extraplanar USG group. Patient demographics and operative characteristics were compared, acknowledging the baseline stone size imbalance as a potential confounder.

**Results:**

Fifty-one (56.0%) patients received extraplanar USG-guided PCNL. The median maximum stone diameter in the conventional group was significantly larger (30 vs. 22 mm, p = 0.001). All tracts were established successfully. Despite larger stones, the conventional group had significantly longer operative times (145 vs. 108 min, p = 0.001). No significant difference was observed in stone-free rates (55% vs. 57%). The extraplanar group showed significantly lower postoperative serum creatinine levels (80 vs. 87 μmol/L, p = 0.03) and shorter hospital stays (8 vs. 10 days, p = 0.01). Postoperative fever occurred in four patients in the extraplanar group (8% vs. 0%, p = 0.07).

**Conclusions:**

Multi-tract PCNL performed under extraplanar USG guidance is safe and efficacious for CRS management. The technique optimizes the puncture strategy, offering superior operative efficiency and potential nephron preservation despite baseline stone load differences.

## Introduction

The incidence of urolithiasis is increasing globally, influenced by factors such as gender, age, diet, and geography, leading to various types of stones with distinct clinical features (1, 2). Complex renal stones (CRS) are typically large (> 2 cm), dense (≥ 950 Hounsfield units), and often associated with hydronephrosis. These stones may also be located across multiple calyces (3). According to the European Association of Urology (EAU) guidelines, CRS are classified as complete or partial staghorn stones, which involve both the renal pelvis and calyces (4, 5). CRS are challenging to manage due to their size and potential morbidity.

Percutaneous nephrolithotomy (PCNL) remains the first-line treatment for stones ≥ 2 cm (6). Over time, advances in technique, instrumentation, and patient positioning have improved stone clearance and minimized complications (7). PCNL success depends on establishing a percutaneous tract, which can be achieved using ultrasonography (USG) or fluoroscopy (8). USG is preferred due to its cost-effectiveness, absence of radiation, and high success rate when performed by skilled urologists (9). Refining USG-guided access is crucial, especially when multiple tracts are needed for complex stone burdens.

Conventional USG-guided puncture involves inserting the needle along the probe’s width, resulting in oblique puncture paths that can increase renal parenchymal injury and bleeding risk. Additionally, real-time needle visibility is limited, particularly in complex renal anatomy or during respiratory motion.

In contrast, we previously described an extraplanar technique where the needle is inserted at the midpoint of the probe’s length, offering a shorter and more vertical path to the renal cortex (10). This technique may reduce parenchymal trauma and improve efficiency, especially in multi-tract cases. The aim of this study is to evaluate the safety and efficacy of extraplanar USG-guided multi-tract PCNL for CRS, hypothesizing that it will reduce operative time and hospital stays while achieving comparable stone-free rates and maintaining a high safety profile.

## Materials and methods

### Study design and patient population

Between May 2017 and December 2020, patients diagnosed with complex renal stones (CRS) and treated with multi-tract percutaneous nephrolithotomy (PCNL) at the University of Hong Kong – Shenzhen Hospital were included in this study. CRS were defined as stones that are typically large (> 2 cm), dense (≥ 950 Hounsfield units), and often associated with moderate-to-severe hydronephrosis, including complete or partial staghorn stones involving both the renal pelvis and calyces.

Patients were excluded if they met any of the following criteria: (1) additional urological conditions (e.g., ureteral calculi, renal abscess); (2) underlying coagulation or cardiopulmonary dysfunction; (3) anatomical abnormalities of the urinary system.

The choice between conventional and extraplanar USG guidance was at the discretion of the operating surgeon based on their assessment of patient anatomy and stone complexity. All procedures were performed by the same specialized urological team to minimize inter-surgeon variability.

This study was conducted in accordance with the ethical principles of the Declaration of Helsinki (11). All participants were informed about the study’s nature, procedures, and risks, and written informed consent was obtained. The study was approved by the institutional review board and ethics committee (Ref (2025):013), and conducted in accordance with relevant guidelines, including the Strengthening the Reporting of Observational Studies in Epidemiology (STROBE) guidelines (12).

### Clinical data

Clinical data were collected from electronic medical records, including age, gender, preoperative urine culture results, and pre- and post-operative laboratory results (hemoglobin [Hb] and serum creatinine [sCr] levels). Positive urine cultures were treated with appropriate antibiotics. Preoperative computed tomography (CT) scans were reviewed to assess stone size, number, location, and morphology.

### Study outcomes

The primary outcome of this study was the stone-free rate (SFR), defined as the absence of stone or clinically insignificant residual stone fragments (< 4 mm in diameter) on the first postoperative CT scan (13).

Secondary outcomes included the operative time, hospital stay, and perioperative complications, such as bleeding and kidney injury. These were evaluated based on serum creatinine (sCr) and hemoglobin (Hb) levels, as well as the incidence of postoperative fever, which was classified as a complication.

### Infection protocol

Patients with positive preoperative urine cultures or febrile urinary infections were administered appropriate antibiotics based on culture sensitivity and allergy history. Per institutional protocol, prophylactic antibiotics were generally limited to 48 hours postoperatively for patients without infectious complications.

### Extraplanar ultrasound technique and multi-tract PCNL

Under general anesthesia, patients were initially placed in the lithotomy position. A 6 French (Fr) ureteral catheter was introduced to the ipsilateral upper urinary tract under rigid ureteroscopic guidance, positioning it beneath the renal stone. The patient was then carefully moved to the prone position with the aid of a transfer belt to allow for slower infusion rates of saline through the ureteral catheter, inducing artificial hydronephrosis for improved renal imaging. Preoperative CT scans were used to determine the optimal number of PCNL access tracts, which were then established under USG guidance.

**Figure 1:**
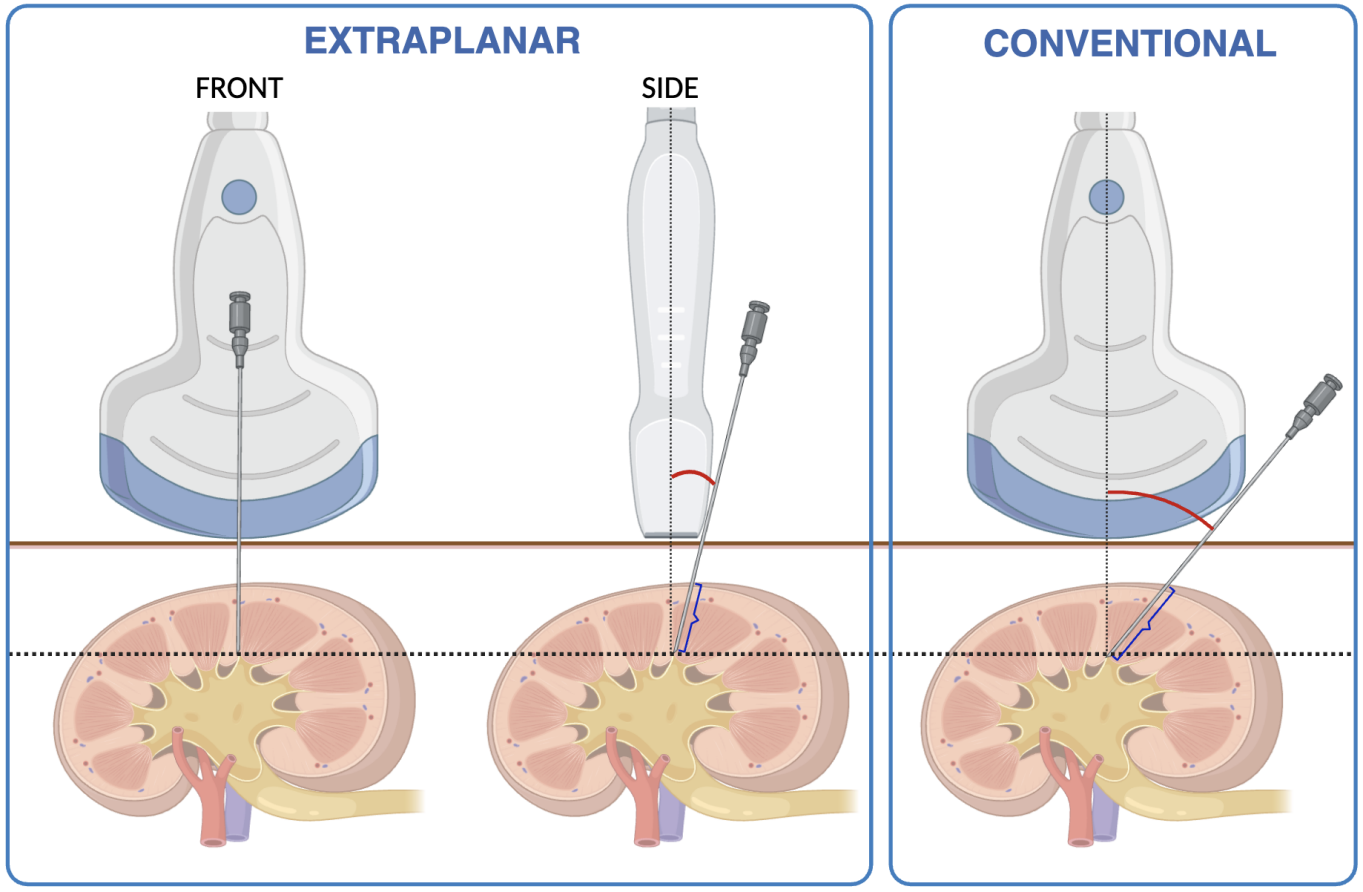
Illustration comparing between extraplanar and conventional ultrasound-guided percutaneous entry shows a much smaller angle for access and needle travelling distance.

The C251 Curved Array transducer and ALOKA ARIETTA60 (Hitachi Medical Corp., Japan) were used. The probe was placed above the target calyx (typically at the 10th or 11th intercostal space, at the intersection of the mid- and posterior-axillary lines). A puncture point was marked at the midpoint of the probe’s length, corresponding to the shortest vertical distance for access (**Figure 1**). An 18 Fr PCNL needle was inserted against the lateral aspect of the probe, requiring a 10°–15° angle for access, and advanced 1–3 cm beyond the renal cortex. Successful access was confirmed by clear urine once the needle stylet was withdrawn.

A J-tip coaxial guidewire was inserted through the access needle, and the needle was withdrawn over the guidewire. A skin incision was made, and tract dilatation was performed using sequential dilators up to 18 Fr or 20 Fr, followed by a matching peel-away sheath for simultaneous stone manipulation. The second and third tracts were established in a similar manner. Finally, nephroscopy was performed with a pneumatic ballistic lithotripsy system for stone removal, and USG was used to confirm the absence of significant residual stones. The procedure was completed by placing a 5 Fr double-J stent and nephrostomy tubes for drainage.

All procedures were performed by a single, specialized urological team with extensive experience in conventional USG-guided PCNL. Mastery of the extraplanar technique required specific training on the 10°–15° lateral entry angle and real-time monitoring of the needle tip as it passed through the calyceal fornix into the collecting system. Based on our institutional experience, we estimate that approximately 20–30 cases are required for a proficient PCNL surgeon to stabilize their proficiency with the extraplanar approach.

### Statistical analysis

Statistical analysis was performed using SPSS software (Version 28.0; IBM Corporation, Armonk, NY). Descriptive statistics were used to summarize patient characteristics. Categorical variables are presented as frequencies and percentages and were compared using the Pearson χ^2^ test or Fisher’s exact test, as appropriate. Continuous variables were assessed for normality using the Kolmogorov-Smirnov test. Normally distributed variables are presented as mean ± standard deviation and were compared using Student’s t test. Non-normally distributed variables are presented as median (25th–75th percentile) and were compared using the Mann-Whitney U test. A two-sided p value < 0.05 was considered statistically significant.

## Results

### Patient demographics and stone characteristics

A total of 91 patients were enrolled in this study, with 51 patients (69% male, mean age 48 ± 12 years) in the extraplanar USG group and 40 patients (60% male, mean age 52 ± 12 years) in the conventional USG group. Baseline characteristics were comparable between the groups. The median maximum stone diameter was significantly larger in the conventional USG group (30 mm, interquartile range [IQR] 21–45 mm) compared to the extraplanar USG group (22 mm, IQR 17–29 mm, p = 0.001) (**Table 1**).

**Table 1:** Comparison of clinical characteristics in patients with complex renal stones who underwent multi-tract PCNL with extraplanar vs. conventional ultrasound guidance.

Parameters	Conventional	Extraplanar	χ^2^ or t/z	*p* value
Total, *n*	40	51	—	—
Male gender, *n* (%)	24 (60)	35 (69)	0.73	0.391
Mean age ± SD, years	52 ± 12	48 ± 12	1.56	0.120
Positive urine culture, *n* (%)	9 (23)	6 (12)	1.87	0.172
Max. stone diameter, mm	30 (21, 45)	22 (17, 29)	3.33	0.001*
Pre-op sCr, μmol/L	85 (71, 100)	75 (64, 92)	1.53	0.131
Mean pre-op Hb ± SD, g/L	137 ± 16	137 ± 17	0.1	0.931

Data are presented as median (25^th^ percentile, 75^th^ percentile) unless otherwise specified.

USG, ultrasound; SD, standard deviation; Pre-op, preoperative; sCR, serum creatinine; Hb, hemoglobin.

A *p* value marked with * is considered statistically significant.

### Number of tracts established

In the extraplanar USG group, 45 patients (88%) had two tracts, and 6 patients (12%) had three tracts. In the conventional USG group, 33 patients (83%) had two tracts, and 7 patients (17%) had three tracts. No significant difference was observed in the number of tracts established between the groups (p = 0.442) (**Table 2**).

**Table 2:** Comparison of procedural characteristics and perioperative outcomes of multi-tract PCNL with extraplanar vs. conventional ultrasound guidance.

Parameters	Conventional	Extraplanar	χ^2^ or t/z	*p* value
Multi-tract PCNL, *n* (%)				
2 channels	33 (83)	45 (88)	0.6	0.442
3 channels	7 (17)	6 (12_		
Mean operative time ± SD, min	145 ± 38	108 ± 36	4.83	0.001*
Hospital stay, days	10 (8, 13)	8 (6, 15)	2.52	0.010*
Post-op sCr, μmol/L	87 (73, 113)	80 (65, 92)	2.11	0.030*
Post-op Hb, g/L	122 ± 18	129 ± 18	1.85	0.052
Post-op fever, *n* (%)	0 (0)	4 (8)	3.28	0.070
Stone-free rate, %	55	57	0.88	0.651
Stratified SFR by tract number, *n* (%)				
2 channels	19 (57.6)	27 (60.0)	—	—
3 channels	3 (42.9)	2 (33.3)	—	—

Data are presented as median (25^th^ percentile, 75^th^ percentile) unless otherwise specified.

USG, ultrasound; mtmPCNL, multi-tract mini percutaneous nephrolithotomy; SD, standard deviation; Post-op, post-operative; sCR, serum creatinine; Hb, hemoglobin.

A *p* value marked with * is considered statistically significant.

### Operative time

The total operative time was significantly shorter in the extraplanar USG group compared to the conventional USG group (108 ± 36 minutes vs. 145 ± 38 minutes, p = 0.001), demonstrating increased procedural efficiency with the extraplanar technique (**Table 2**).

### Stone-free rate

The overall SFR was similar between the two groups (57% for extraplanar vs. 55% for conventional, p = 0.651). However, when stratified by the number of tracts, the SFR for patients with two tracts was higher in both groups: 60.0% (n = 27/45) for the extraplanar group and 57.6% (n = 19/33) for the conventional group. For patients with three tracts, the SFR was lower: 33.3% (n = 2/6) for the extraplanar group and 42.9% (n = 3/7) for the conventional group. These findings are summarized in **Table 2**.

A learning curve effect was observed in the extraplanar USG group. In the final 30 cases, the SFR improved to 62%, compared to 54% in the earlier phase of the study, suggesting that technical proficiency improved with experience.

### Postoperative fever

Postoperative fever occurred in 4 patients (8%) in the extraplanar USG group, while no cases of fever were reported in the conventional USG group (p = 0.070). All cases of fever in the extraplanar group were resolved with appropriate antibiotic therapy.

### Renal function and hemoglobin levels

The postoperative sCr levels were significantly lower in the extraplanar USG group (80 μmol/L, IQR 65–92) compared to the conventional USG group (87 μmol/L, IQR 73–113, p = 0.03), indicating better nephron preservation with the extraplanar technique. No significant difference in postoperative hemoglobin (Hb) levels was observed between the groups (129 ± 18 g/L vs. 122 ± 18 g/L, p = 0.052).

### Hospital stay

The mean duration of hospital stay was significantly shorter in the extraplanar USG group (8 days, IQR 6–15) compared to the conventional USG group (10 days, IQR 8–13, p = 0.01), suggesting faster recovery with the extraplanar technique.

## Discussion

This retrospective study compared extraplanar versus conventional USG-guided multi-tract PCNL in 91 patients with CRS. The extraplanar technique demonstrated significantly shorter operative times and hospital stays with lower postoperative serum creatinine levels, suggesting improved procedural efficiency and potential nephron preservation. Stone-free rates were comparable between groups despite a baseline stone size imbalance favoring the extraplanar group. The overall complication rate was low, with postoperative fever occurring exclusively but non-significantly in the extraplanar group. These findings suggest that extraplanar USG guidance may offer advantages in operative efficiency and renal safety for multi-tract PCNL, though larger prospective studies are needed to confirm these benefits.

The significantly shorter operative times observed with extraplanar guidance warrant careful interpretation given the baseline stone size imbalance between groups. Despite the conventional group having larger stones that would typically require more extensive fragmentation and extraction time, operative duration remained substantially longer in this group. This paradox suggests that improved access efficiency with extraplanar guidance, including shorter skin-to-cortex distance and fewer needle repositioning maneuvers, may compensate for stone volume challenges. Future studies incorporating quantitative stone burden measures and stratified analyses are needed to isolate the independent effect of access technique on operative efficiency.

Our SFRs must be contextualized within the complexity of treated cases and methodological rigor. All patients had CRS with hard staghorn calculi or multi-calyceal stones, and we employed strict criteria requiring absence of fragments > 4 mm on first postoperative day CT rather than less sensitive imaging modalities. Published SFRs for PCNL monotherapy in staghorn stones range from 49% to 78%, positioning our results within established norms for such challenging cases (14). The stratification by tract number revealed an expected pattern: lower clearance rates with three tracts likely reflect greater stone complexity rather than technique limitations, as the need for additional tracts inherently indicates more challenging anatomy and stone distribution (3). The observed learning curve effect, with improved outcomes in later cases, underscores the importance of technical proficiency in optimizing results with novel access techniques. Based on institutional experience, approximately 20–30 cases are required for proficient PCNL surgeons to master the lateral entry angle control and real-time needle tip monitoring specific to this approach.

Safety considerations are paramount in multi-tract PCNL given the cumulative parenchymal trauma. Our complication rate was substantially lower than the reported average for PCNL (15, 16). While standard PCNL with larger tracts may achieve marginally higher stone-free rates, we prioritized miniaturized tracts to reduce bleeding risk, a strategy supported by meta-analyses demonstrating comparable efficacy with improved safety profiles (17, 18). The significantly lower postoperative serum creatinine in the extraplanar group merits particular attention, suggesting that more precise tract creation with reduced intraparenchymal traversal may offer superior nephron preservation. This finding aligns with prior work indicating that although tract-related renal damage represents a small fraction of cortical surface (19), technique refinements may still yield measurable functional benefits.

The observation of postoperative fever exclusively in the extraplanar group, while not statistically significant, deserves consideration. With comparable baseline infection risks and similar tract numbers between groups, the low event rate suggests this distribution may be attributable to chance. However, we cannot exclude procedural contributors related to the learning curve, such as variations in puncture angle control or transient intrarenal pressure changes. The small sample size and low overall incidence preclude definitive conclusions; larger multicenter studies with standardized infection protocols are warranted to clarify whether this technique influences infectious outcomes.

Several limitations merit acknowledgment. The retrospective design and baseline stone size imbalance introduce potential confounding, though surgeon consistency across all procedures minimizes technique-related variability. The modest sample size limited statistical power for rare events. Long-term outcomes including renal function trajectory, stone recurrence, and late complications remain unknown. Prospective randomized trials incorporating standardized stone complexity metrics and extended follow-up are needed to validate these findings.

## Conclusion

Extraplanar USG-guided multi-tract PCNL demonstrates promise as a safe and efficient approach for managing CRS, with potential advantages in operative efficiency and nephron preservation. Validation through larger prospective studies with long-term follow-up is necessary to establish its role in contemporary endourology practice.

## Data Availability

The raw data supporting the conclusions of this article will be made available by the authors, without undue reservation.
